# The Effect of Traditional Chinese Medicine on Neural Stem Cell Proliferation and Differentiation

**DOI:** 10.14336/AD.2017.0428

**Published:** 2017-12-01

**Authors:** Wei Qin, Shiya Chen, Shasha Yang, Qian Xu, Chuanshan Xu, Jing Cai

**Affiliations:** ^1^Academy of Integrative Medicine, Fujian University of Traditional Chinese Medicine, Fuzhou 350122, China; ^2^College of Integrative Medicine, Fujian University of Traditional Chinese Medicine, Fuzhou 350122, China; ^3^School of Chinese Medicine, Faculty of Medicine, The Chinese University of Hong Kong, Shatin, Hong Kong

**Keywords:** Neural stem cells, proliferation, differentiation, traditional Chinese medicine

## Abstract

Neural stem cells (NSCs) are special types of cells with the potential for self-renewal and multi-directional differentiation. NSCs are regulated by multiple pathways and pathway related transcription factors during the process of proliferation and differentiation. Numerous studies have shown that the compound medicinal preparations, single herbs, and herb extracts in traditional Chinese medicine (TCM) have specific roles in regulating the proliferation and differentiation of NSCs. In this study, we investigate the markers of NSCs in various stages of differentiation, the related pathways regulating the proliferation and differentiation, and the corresponding transcription factors in the pathways. We also review the influence of TCM on NSC proliferation and differentiation, to facilitate the development of TCM in neural regeneration and neurodegenerative diseases.

Both embryonic and adult neural stem cells (NSCs) are widely distributed in the nervous system. Embryonic NSCs are widely distributed in the brain, while adult NSCs are mainly distributed in the subventricular zone (SVZ) of the lateral ventricle wall and the subgranular zone (SGZ) of the hippocampus dentate gyrus [1, 2]. NSCs go through different stages of neural progenitor cells (NSPCs), neural precursor cells (NPCs), and neuroblasts in the process of proliferation and differentiation into neural cell lineage [3-7]. In the research on NSCs, their identification and differentiation in these stages are related to whether they can be accurately induced, differentiated, and migrated. The corresponding markers of NSCs at different stages have been identified and confirmed, and are summarized in this study.

NSCs can differentiate into different types of neural cells under specific conditions, which can provide new methods of cerebral injury repair and neurological disease treatment. Numerous studies have shown that NSCs have a therapeutic effect on nervous system injuries and degenerative diseases such as Alzheimer’s disease (AD), Parkinson's disease (PD), spinal injury, amyotrophic lateral sclerosis (ALS), vascular dementia, cerebral hemorrhage, and Huntington’s disease [8-17]. However, the differences between animal models and human diseases mean that the clinical application of stem cell therapy is still some way off.

Traditional Chinese medicine (TCM) has the general advantages of multi-targets, multi-levels, and multi-paths [18-21]. It can regulate NSC proliferation and differentiation by changing the microenvironment of NSCs and indirectly regulating endogenous and exogenous factors. Studies have shown that single herbs, herb extracts/Chinese herbal monomers and compounds, and Chinese medicinal preparations have, to some extent, a role in regulating NSC proliferation and differentiation [22-27].

**Table 1:** Markers of stem cell proliferation and differentiation in different stages.

Marker	Property	Affected cell type	Function	Refs.
Hopx	atypical homeodomain only protein	NSCs	regulates hippocampal neurogenesis by modulating Notch signaling	[33]
Hes3	basic helix-loop- helix gene	NSCs	promote the proliferation of NSCs maintain the undifferentiated state of NSCs	[34, 35]
TRIP6	zyxin family proteins	NSCs	promote the self-renewal and proliferation of NSCs	[36]
CycE	cyclin	NSCs	regulate neurogenesis in the adult hippocampus	[37]
JAM-C	surface protein	NSCs	maintain the pluripotency of NSCs	[38]
PtdGlc	lipid	NSCs	identify, isolate, and differentiate NSCs	[39]
CD9	transmembrane protein	NSPCs	have an impact on the cell adhesion, migration, proliferation and differentiation	[40, 41]
CD15	transmembrane protein	NSPCs	promote the survival of NSCs; promote the differentiation of NSCs into oligodendroglia	[40, 42]
CD81	transmembrane protein	NSPCs	control the cell migration	[40, 43]
S100β	acid calcium binding protein	NSPCs	regulate the proliferation of NSCs	[44]
CD133	transmembrane protein	NPCs	promote the expansion of NSCs *in vitro* and its degree of specialization	[45, 46]
CD24	transmembrane protein	NPCs	play an important role in self-renewal; maintain NSCs	[45, 47, 48]
Pax2	paired box gene	NPCs	regulate the migration and proliferation of nerve cells	[49]
NG2	transmembrane proteoglycan	NPCs	regulate the migration of the oligodendrocyte precursor cells	[50, 51]
Nestin	intermediate filaments protein cytoskeletal protein	NSCs and NSPCs	be a marker for proliferating or migrating cells; participate in cytoskeleton formation; remodel cells along with other structural proteins;	[52-55]
Musashi1	RNA- binding protein	NSCs and NSPCs	determine the fate of stem cells; maintain the undifferentiated state of NSCs or NSPCs;	[56-61]
Tub-II	cytoskeleton protein	NSCs and NSPCs	reflect the structural changes in the development of the brain	[62, 63]
SOX2	high-mobility group proteins	NSCs and NSPCs	play a role in self-renewal and maintenance of NSCs; prevent the apoptosis of NSCs.	[56, 64, 65]
SOX1	high-mobility group proteins	NSCs and NSPCs	promote the self-renewal of NSCs	[66-68]
Sp8	zinc finger protein	NSCs and NSPCs	maintain the undifferentiated state of NSCs	[69, 70]
S100A6i	low-molecular-weight calcium-binding proteins	NSCs and NPCs	promote the neurogenesis in the hippocampus; play an important role in the differentiation and maturation of astrocytes	[71]
Prox1	homeobox transcription factor	neuroblasts	play an important role in regulating the proliferation and differentiation of NSCs; maintain the intermediate progenitor cells	[72, 73]
Cyc D1	cyclin	neuroblasts	promote the proliferation of NSCs; inhibit their differentiation	[74, 75, 204]
DCX	microtubule-associated protein	neuroblasts	regulate the migration of neural cells	[76, 77]

The table lists the markers of NSCs proliferation and differentiation, the property of the markers, the cell type they affect, and their function in NSC proliferation and differentiation: Tub-II, tubulin beta II; SOX2, sex-determining region Y-box2; SOX1, sex-determining region Y-box1; Sp8, specificity protein 8; PAX2, paired box protein 2; Hopx, homeodomain only protein X; Hes3, hairy and enhancer of split 3; TRIP6, thyroid receptor-interacting protein 6; CycE, cyclinE;JAM-C, junctional adhesion molecule-C; PtdGlc, phosphatidylglucoside; S100β, S100 calcium-binding protein B; NG2, Neuron glia antigen 2; Cyc D1, Cyclin D1; Prox1, prospero homeobox protein 1; DCX, doublecortin; NSCs, neural stem cells; NSPCs, neural progenitor cells; NPCs, neural precursor cells.

In this article, we discuss the regulation of NSC proliferation and differentiation by the relevant pathways and the target genes corresponding to the pathways, and review the effects of TCM on the proliferation and differentiation of NSCs, to further develop the study of NSC proliferation and differentiation by TCM.

**Table 2:** The main transcription factors and associated signaling pathways in NSC proliferation and differentiation.

TFs	Protein family	Pathway	*In vivo* or *in vitro*	Effect on NSCs	Affected cell type	Location of expression	Refs.
Hes1	bHLH	Notch	*In vivo* and in *vitro*	Play a role in maintenance of NSCs; Inhibit the differentiation of NSCs into neurons; Have an effect on the maintenance and self-renewal of NSPCs	NSCs and NSPCs	SVZ, SGZ	[81-87]
Hes5	bHLH	Notch	*In vitro*	promote the proliferation of NSCs	NSCs and NSPCs	SVZ	[88-90]
Mash1	bHLH	Notch	*In vivo* and in *vitro*	Promote the differentiation of NPCs	NSPCs and NPCs	SVZ, SGZ	[91-96]
NeuroD	bHLH	Notch	*In vivo* and in *vitro*	Determine the fate and differentiation of cells; Determine the survival of neurons	NSPCs and NPCs	SGZ, SVZ, VZ	[97-102]
zfp488	ZFP	Notch	*In vivo*	Promote the differentiation of NSCs into the oligodendrocytes	NSCs and NSPCs	SVZ	[103, 104]
Ngn1	bHLH	Notch	*In vivo*	Promote neurogenesis; Play a specific role in the maintenance of NSPCs; Promote the differentiation of NPCs *in vivo*	NSPCs and NPCs	SVZ	[105, 106]
Ngn2	bHLH	Notch	*In vivo* and in *vitro*	Play a regulatory role in neurogenesis; Control the balance of the maintenance and differentiation of NSPCs	NSPCs	VZ, SVZ	[107, 108]
Fezf2	ZFP	Notch	*In vivo* and in *vitro*	Has a role in the maintenance and differentiation of NSCs	NSCs, NSPCs and NPCs	SVZ, VZ	[109, 110]
Hey1	bHLH	Notch	*In vivo*	Play a role in the maintenance of NSCs	NPCs	VZ, SVZ	[111-113]
Gsx2	HOM	Notch	*In vivo*	Reduce the ability of NSCs to proliferate and self-renew; Reduce the transformation of NSCs into neurons and glial cells	NSCs and NSPCs	SVZ, VZ	[114-116]
Pax6	HOM	Wnt	*In vivo* and in *vitro*	Control the balance of the maintenance and differentiation of NSCs; Play an important role in maintenance, self-renewal and multi-directional differentiation of NSCs	NSCs and NSPCs	SVZ, OB, SGZ, VZ	[121-126]
Emx2	HOM	Wnt	*In vivo* and in *vitro*	Control the proliferation and migration of NPC	NSCs and NPCs	SVZ, VZ	[127-129]
Dix2	HOM	Wnt	*In vivo* and in *vitro*	Promote the neurogenesis and proliferation	NPCs	SVZ, OB	[130, 131]
Pax3	HOM	Wnt	*In vivo* and in *vitro*	Regulate the differentiation of NSCs; Determine the fate of cells; Maintain the undifferentiated state of NSCs.	NSPCs	VZ	[132-137]
Oct4	POU	Wnt	*In vivo* and in *vitro*	Play an important role in the maintenance of the pluripotency of NSCs; Promote the proliferation and self-renewal of NSCs	NSCs	SVZ	[138-140]
Prox1	HOM	Wnt	*In vivo* and in *vitro*	Promote the proliferation of NSCs; Play an important role in the maintenance of intermediate progenitor cells	NSPCs	SGZ	[141, 142]
Nkx2.2	HOM	Shh	*In vivo*	Promote the differentiation of oligodendrocytes	NSPCs and NPCs	SVZ, OB	[152-156]
Gli-1	ZFP	Shh	*In vivo* and in *vitro*	Promote the proliferation of NPCs	NPCs	SVZ, SGZ	[158-162]
Sox2	HMG	BMP	*In vivo* and in *vitro*	Play a role in self-renewal and maintenance of NSCs; Prevent the apoptosis of NSCs	NPCs and NSCs	SVZ, SGZ, VZ	[167-172]
Olig2	bHLH	BMP	*In vivo* and in *vitro*	Induce the differentiation of NSCs into oligodendrocytes; Promote the maturation of the differentiated cells.	NSPCs	SVZ	[173-175]

The table lists the main transcription factors and associated signaling pathways in NSC proliferation and differentiation, the protein family the transcription factors belong to, the cell type, they influence and their effect on NSCs proliferation and differentiation, and the main location of their expression. Hes1, hairy, and enhancer of split 1; Hes5, hairy and enhancer of split 5; Mash1, achaete-scute homolog 1; NeuroD, neurogenic differentiation factor-6; zfp488zinc finger protein 488; Ngn1,Neurogenin1; Ngn2, Neurogenin2; Fezf2, forebrain embryonic zinc finger 2; Gsx2, GS Homeobox 2; Hey1, hairy/enhancer-of-split related with YRPW motif protein 1; Pax6, paired box protein 6; Pax3, paired box protein 3; Emx2, empty spiracles homeobox 2; Dix2, distal-less homeobox 2; oct4, octamer-binding transcription factor 4; Olig2, oligodendrocyte lineage transcription factor 2; Nkx2.2,NK2 homeobox 2;Gli-1, glioma associated oncogene-1; bHLH, basic helix-loop-helix; HOM, homedomain; HMG, high mobility group; PC, Polycomb; ZFP, zinc finger proteins; BMP, bone morphogenetic protein; Shh, sonic hedgehog; SVZ, subventricular zone; SGZ, dentate gyrus subgranular zone; VZ, ventricular zone; OB, olfactory bulb.

## 1. The stages and related markers of NSC proliferation and differentiation

NSCs go through different stages of NSPCs, NPCs, and neuroblasts in the process of proliferation and differentiation into mature neural cells [28-32]. NSCs can express specific molecular markers in the proliferation and differentiation stages. These markers have the function of selectively binding to signal molecules and are involved in the expression of cell signaling. In addition, transcription factors and cell adhesion molecules are significant for the differentiation of NSCs. The Nestin, Musashi1, sex-determining region Y-box2 (SOX2), Prox1, and CD family proteins are the most common markers.

An increasing number of novel markers have also been used to identify NSCs. These include homeodomain-only protein X (Hopx) [33], hairy and enhancer of split 3 (Hes3) [34, 35], thyroid receptor-interacting protein 6 (TRIP6) [36], Cyclin E (CycE) [37], junctional adhesion molecule-C (JAM-C) [38], and phosphatidylglucoside (PtdGlc) [39], which are mainly expressed in NSCs and can be used as characteristic markers. The expression of CD9 [40, 41], CD15 [40, 42], CD81 [40, 43], and S100 calcium-binding protein B (S100β) [44] are commonly expressed in NSPCs. The same CD family members CD133 [45, 46] and CD24 [45, 47, 48], paired box protein 2 (Pax2) [49] of the paired box gene family, and transmembrane proteoglycan (NG2) [50, 51] are mainly expressed in NPCs. Nestin [52-56], Musashi1 [56-61], cytoskeleton protein (Tub-II ) [62, 63], sex-determining region Y-box2 (SOX2) [56, 64, 65], sex-determining region Y-box1 (SOX1) [66-68], specificity protein 8 (Sp8) [69, 70], and low-molecular-weight calcium-binding proteins (S100A6i ) [71] are expressed in both NSCs and NSPCs, while prospero homeobox protein 1 (Prox1) [72, 73], Cyclin D1 (CycD1) [74, 75], and doublecortin (DCX) [76, 77] are expressed in the neuroblast. The properties of the related markers, the stage of the labeled cells, and the function of the markers are summarized in Table 1.

## 2. Signaling pathways and major transcription factors involved in NSC proliferation and differentiation

Multiple signaling pathways regulate the process of NSCs proliferating and differentiating into mature neurons. These pathways determine the fate of NSCs by regulating the expression and activity of different transcription factors, and many pathways are involved. The Notch, Wnt, bone morphogenetic protein (BMP), and sonic hedgehog (shh) signal pathways have been most studied. Downstream of the signal pathway are usually target genes that can be used as transcription factors to regulate the process of proliferation, differentiation, and migration of NSCs, and different signaling pathways and transcription factors can act synergistically to regulate this process. These are summarized in Table 2.

### 2.1 The Notch signaling pathway

The Notch gene was originally found by Morgan and colleagues in drosophila in 1917 [78]. The partial deletion of this gene function was found to lead to a gap in the wing edge of drosophila. The Notch signaling pathway is a highly conserved pathway, which is widespread in invertebrates and mammals, and it determines the fate of cells by precisely regulating cells growth, differentiation, and apoptosis [79]. Numerous studies have shown that the Notch signaling pathway plays an important role in the proliferation and differentiation of NSCs, particularly for maintaining an undifferentiated state and the ability for self-renewal [80]. The common target genes of the pathway are the hairy and enhancer of split 1 (Hes1), hairy and enhancer of split (Hes5), achaete-scute homolog 1 (Mash1), neurogenic differentiation factor-6 (NeuroD), zinc finger protein 488 (zfp488), Neurogenin1 (Ngn1), Neurogenin2 (Ngn2), forebrain embryonic zinc finger 2 (Fezf2), GS Homeobox 2 (Gsx2), hairy/enhancer-of-split related with YRPW motif protein 1 (Hey1), etc. These transcription factors play an important role in the regulation of NSCs proliferation and differentiation. Hes1, Hes5, Mash1, NeuroD, zfp488, Ngn1, Ngn2, and Hey1, are members of the basic helix-loop-helix (bHLH) gene family, which can play a role in the regulation of NSCs *in vivo* and *in vitro*. Hes1 is mainly expressed in the SVZ. It can maintain the state of NSCs, inhibit their differentiation into neurons, and also have an effect on the maintenance and self-renewal of NSPCs [81-87]. Hes5 can promote the proliferation of NSCs, which is mainly expressed in SVZ [88-90]. Mash1 is a target gene of the Notch signaling pathway, which can be expressed in SVZ and SGZ during neurogenesis. Mash1 is also a determinant for the differentiation and maturation of neural *in vivo* and *in vitro*. Studies have shown that Mash1 promote the differentiation of NSPCs and NPCs [91-96]. NeuroD, a member of the bHLH gene family, can determine the fate and differentiation of cells. It is mainly expressed in SGZ, which regulate neurogenesis *in vivo* and *in vitro* [97-102]. zfp488 promotes the differentiation of NSCs into oligodendrocytes [103, 104]. Ngn1 and Ngn2 are the other two bHLH family genes, and Ngn1 can promote neurogenesis and the differentiation of NPCs *in vivo*. It also plays a specific role in the maintenance of NSPCs [105, 106]. Ngn2 can be expressed in SVZ and the ventricular zone (VZ) during neurogenesis, and plays a regulatory role in neurogenesis *in vivo* and *in vitro*. Ngn2 also controls the balance between the maintenance and differentiation of NSPCs [107, 108]. Fezf2 is a zinc finger transcription factor, and research has shown that it can promote the differentiation of NSCs in SVZ. It also influences the maintenance of NSCs [109, 110]. Hey1 has a maintenance effect on NSCs, which is mainly expressed in VZ and SVZ [111-113]. Gsx2 is a homeodomain transcription factor, which is mainly expressed in SVZ. Gsx2 plays an important role in the inhibition of neurogenesis. For example, Gsx2 can reduce the proliferation and self-renewal of NSCs, and inhibit the differentiation of NSCs into neurons and glial cells. Thus, the NSCs and NSPCs can be maintained in a static and undifferentiated state [114-116].

### 2.2 Wnt signaling pathway

The Wnt signaling pathway is named after its promoter protein Wnt, which is synthesized by the wingless gene of the African drosophila and the proto-oncogene Int1 of the mouse. The four main Wnt signal pathways are the canonical Wnt/β-catenin pathway, the Wnt/polarity pathway, the Wnt/Ca^2+^ pathway, and the intracellular pathways that regulate spindle orientation and asymmetric cell division [117]. Of these, the canonical Wnt/β-catenin signaling pathway is important in regulating the proliferation and differentiation of NSCs [118-120]. The target genes regulated by the Wnt signaling pathway are paired box protein 6 (Pax6), paired box protein 3 (Pax3), empty spiracles homeobox 2 (Emx2), distal-less homeobox 2 (Dix2), Octamer-binding transcription factor 4 (Oct4), Prox1, etc. Pax6, Emx2, Dix2, Pax3, and Prox1 belong to the zinc finger transcription factor. Pax6 regulates the proliferation and differentiation of NSCs *in vivo* and *in vitro*. Studies have shown that Pax6 can be expressed in both SVZ and OB, and can control the balance between the self-renewal and differentiation of NSCs, and is important in their maintenance, self-renewal, and multi-directional differentiation [121-126]. Emx2 is a target gene regulated by the Wnt signaling pathway, which regulates neurogenesis *in vivo* and *in vitro*. Emx2 mainly affects NPCs through controlling the migration and differentiation of NPCs [127-129]. Dix2 is expressed in SVZ and OB, and regulates the proliferation of NPCs in SVZ [130, 131]. Pax3 is a DNA-binding protein, mainly expressed in VZ, and plays a role in the maintenance of NSPCs. The overexpression of Pax3 can inhibit the differentiation of NSCs. But if it is inhibited, it will promote their differentiation [132-137]. Oct4 belongs to the POU protein family, and can be expressed *in vivo* and *in vitro*. Oct4 is important in the maintenance of pluripotent stem cells, and promotes the proliferation and self-renewal of NSCs [138-140]. Prospero homeobox protein 1 (Prox1) plays an important role in the maintenance of NSPCs and regulates the differentiation of NSCs in SGZ [141, 142].

### 2.3 Shh signaling pathway

The hedgehog gene was first found in Drosophila in 1980, and has three homologous genes: sonic hedgehog (Shh), Indian hedgehog (Ihh), and desert hedgehog (Dhh). They encode Shh, Ihh, and Dhh proteins, respectively [143-145]. The Sonic hedgehog is an important developmental regulatory factor produced by the notochord during embryonic development [146]. Shh is important in regulating the migration, survival, and proliferation of NSCs [147-149]. The Shh signaling pathway can regulate the self-renewal of NSCs by increasing their symmetrical division [150, 151]. NK2 homeobox 2 (Nkx2.2) is an important transcription factor involved in the regulation of the Shh pathway. Nkx2.2 can be expressed in both SVZ and OB, and can promote the differentiation of oligodendrocytes and inhibit their self-renewal ability [152-157]. Glioma-associated oncogene-1 (Gli-1) is a member of the ZFP protein family and a target gene of the Shh pathway, which can be expressed *in vivo* and *in vitro* and promote the proliferation of NPCs [158-162].

**Table 3:** Effects of single herb and compound Chinese medicinal preparations on NSC proliferation and differentiation.

Classification	TCM	Affected cell type	Effect on NSCs	Main mechanisms	*In vivo* or in *vitro*	Refs.
Compound Chinese medicinal preparations	BuyangHuanwu Decoction	NSCs and NSPCs	Promote the proliferation and differentiation of NSCs and NSPCs	Decrease the content of Ca^2+^ in the cells, and increase the expression of NF and GFAP.	*In vivo* and *in vitro*	[177-179]
	Jiawei Sini San	NPCs	Promote the proliferation of NPCs and inhibit apoptosis	The expression of nestin, beta-tubulin-III, and fibrillary acidic protein glial were significantly increased	*In vitro*	[180]
	Shengyu decoction	NSCs and NSPCs	Promote the proliferation of NSCs/NSPCs and their differentiation into neurons	Increase the expression of TN-C, GDNF, NCAM, and NGF, and inhibits the expression of Nogo-A	*In vivo*	[181]
	FuzhiSan	NSPCs	Promote the proliferation of NSPCs; Improve the survival rate of newborn cells	Promote the neurogenesis in hippocampus	*In vivo*	[182]
	XiehuoBushenDecocfion	NSCs	Promote the survival and differentiation of NSCs	Enhance the expression of IL-4 mRNA, and down-regulate the expression of IFN-gama mRNA	*In vivo* and *in vitro*	[27]
	PMC-12	NSPCs	Promote the proliferation of NSPCs in the hippocampus; Improve the survival rate of newborn nerve cells	Increased levels of BDNF, p-CREB and synaptophysin	*In vivo*	[183]
Single herb	Salvia miltiorrhiza Bge	NSCs	Promote the differentiation of induced multifunctional NSCs into neurons *in vitro*; Promote the survival, collection and differentiation of NSCs derived from multifunctional stem cell	Increase the expression of nestin and MAP2	*In vivo* and in *vitro*	[22]
	Sambucus williamsii Hance	NSCs	Promotethe differentiationof NSCs into neurons	Up-regulate the expression of Tuj1 and nestin genes, and down-regulate the expression of Oct4 and Sox2 genes	*In vitro*	[184]
	Scutellariacalensis Georgi, Phellodendronchinense Schneid, Ligusticumwallichii Franch	NSCs and NPCs	Promote the proliferation of NSCs and NPCs	Modulate HPA axis and increase the content of corticosterone	*In vivo* and *in vitro*	[23]

The table lists effects of single herb and compound Chinese medicinal preparations on the NSC proliferation and differentiation, and underlying mechanism, and the cell type they affected; PMC-12, polygonummultiflorum Thunberg complex composition-12; Tuj1, tubulin-1; MAP2, microtubule-associated protein 2; HPA, hypothalamus-pituitary-adrenal; NF, neurofilament; GFAP, glial fibrillary acidic protein; TN-C, Tenascin-C; GDNF, glial cell line-derived neurotrophic factor; NVAM, neural cell adhesion molecule; NGF, Nerve growth factor; BDNF, brain derived neurotrophic factor; p-CREB, phosphorylated cAMP-response element binding protein.

**Table 4:** Effects of active components of Chinese herbs on NSC proliferation and differentiation.

Effective components of Chinese herbs	Origin	Categories of Chinese herbs	Affected cell type	Effects	Underlying mechanisms	*In vivo* or *in vitro*	Refs.
Ginsenoside Rg1	Panax ginseng C. A. Mey	Tonifying Qi herbs	NSCs and NSPCs	Promote the differentiation of NSCs and NSPCs	Increase the expression of SOX-2 and decrease the expression of IL-1β, IL-6 and TNF-α; Enhance the role of anti-inflammatory and antioxidant	*In vivo*	[186, 187]
Ginsenoside Rd	Panax ginseng C. A. Mey	Tonifying Qi herbs	NSCs	Promote the proliferation of NSCs	Regulate the expression of neurotrophic factor 3 and activate the expression of iNOS and NMDA receptors	*In vivo* and *in vitro*	[188]
Oleanolic acid	Ligustrum lucidum Ait	Tonifying Yin herbs	NSCs and NPCs	Promote the self-renewal and differentiation of NSCs; Promote the neurogenesis in hippocampus	Increase the expression of tubulin and the ratio of tubulin /DAPI	*In vivo* and *in vitro*	[24]
Stilbene glucoside	Fallopia multiflora (Thunb.) Harald	Tonifying blood herbs	NSCs	Promote the self-renewal and differentiation of NSCs	Increase the expression of tubulin and the ratio of tubulin /DAPI	*In vitro*	[24]
Resveratrol	Fructus Mori	Tonifying Yin herbs	NSCs	Promote the survival and proliferation of NSCs	Up-regulate the expression of Ptch-1, Smo, Gli-1 protein and RNA	*In vitro*	[25, 158]
(+)-Cholesten-3-one	Chinemys reevesii (Gray)	Tonifying Yin herbs	NSCs	Induce NSCs into dopaminergic neurons	Activate BMP signal; Improve the expression of TH and BMPR-IB	*In vitro*	[189]
Psoralen	Psoralea corylifolia L.	Tonifying kidney herbs	NSCs	Increase the expression of GFAP protein in NSCs *in vitro*	Increase the expression of GFAP protein	*In vitro*	[190]
Icariin	Epimediumgrandiflorum Morr	Tonifying kidney herbs	NSCs	Promote the self-renewal and differentiation of NSCs	Mediate the related kinase signal transduction pathways	*In vitro*	[191]
Salvianolic acid B	Salvia miltiorrhiza Bge	Huoxuehuayu herbs	NSCs and NSPCs	Maintain the self-renewal of NSCs/NSPCs Promote the proliferation of NSCs	Regulate PI3K/Akt signaling pathway; Improve the expression of tau mRNA; Down-regulate the expression of mRNA GFAP	*In vivo* and *in vitro*	[192, 193]
TMP	Ligusticum wallichii Franch	Huoxuehuayu herbs	NSCs	Promote the proliferation and differentiation of NSCs	Increase the phosphorylation of erk1/2; Reduce the phosphorylation of p38	*In vitro*	[194, 195]
PNS	Panax Notoginseng (Burk.) F.H. Chen	Huoxuehuayu herbs	NSCs	Promote the self-renewal, proliferation, and differentiation of NSCs	Improve the expression of tuj-1, vimentin, and nestin mRNA	*In vitro*	[196]
Bilobalide	Ginkgo biloba	Huoxuehuayu herbs	NSCs	Promote the proliferation of NSCs	Increase the phosphorylation of CREB and the level of the neurotrophic factor	*In vivo*	[197]
Berberine	Coptis chinensis Franch	Qingrejiedu herbs	NSCs	Inhibit cell cycle arrest Promote the survival and differentiation of NSCs	Improve the activity of cell viability-dependent NMDA	*In vivo* and *in vitro*	[198]
Baicalein	Scutellaria baicalensis Georgi	Qingrejiedu herbs	NSCs and NPCs	Promote the differentiation of NPCs into neurons; Inhibit the apoptosis and promote the proliferation of NSCs	Increase the expression of presynaptic protein, synapsin I, and PSD95	*In vivo*	[199, 200]
Baicalin	Scutellaria baicalensis Georgi	Qingrejiedu herbs	NSCs and NSPCs	Determine the fate of NSCs; Promote the differentiation of NSCs and NSPCs	Reduce the expression of p-STAT3 and Hes1; Increase the expression of NeuroD1 and Mash1; Regulate the expressionof p-stat3 and bHLH protein family	*In vivo* and *in vitro*	[201, 202]
Paeoniflorin	Paeonia lactiflora Pall, Paeonia suffruticosa	Qingrejiedu herbs	NSCs and NSPCs	Promote the proliferation of nerve cells and inhibit the apoptosis of cells	Activate the PI3k/Akt-1 signaling pathway	*In vitro*	[203]

The table lists the effects of active components of Chinese herbs on NSC proliferation and differentiation and the related mechanism, the cell type they affected, the Chinese herbs the components are extracted from, and the categories of Chinese herbs, including tonifying “Qi” herbs, blood herbs, “Yin” herbs, “Yang” herbs, and HuoXueHuayu herbs and Qingrejiedu herbs; PNS, panax notoginseng saponins; TMP, tramethylpyrazine; TNF-a, tumor necrosis factor a; iNOS, inducible nitric oxide synthase; NMDA, N-methyl-D-aspartic acid receptor; TH, tyrosine hydroxylase; BMPR-I, bone morphogenetic protein receptor IB; Patched1; Smo, Smoothened; PSD95, postsynaptic density proteins 95.

### 2.4 BMP signaling pathway

Bone morphogenetic protein (BMP) is a type of acidic peptide, and members of the transforming growth factor β (TGF-β) superfamily [163, 164]. BMP is an intercellular signal protein, and is important in the regulation of proliferation, differentiation, and apoptosis of NSCs [165, 166]. The sex-determining region Y-box2 (Sox2) and oligodendrocyte lineage transcription factor 2 (Olig2) are two target genes of the BMP pathway. Sox2 is a high-mobility group box transcription factor gene that can be expressed in the NSCs of SVZ and SGZ, and can also be expressed in the NPCs of VZ. Sox2 can regulate the self-renewal of NSCs and NPCs and prevent the apoptosis of NSCs [167-172]. Olig2 is a helix-loop-helix-transcription factor, mainly expressed in SVZ, and can promote the proliferation of NSPCs, induces the differentiation of NSCs into oligodendrocytes *in vitro,* and promotes the maturation of differentiated cells [173-175].

## 3 Effects of TCM on NSCs proliferation and differentiation

Numerous studies have shown that TCM has a regulatory effect on NSC proliferation and differentiation. TCM can improve the microenvironment, promote neurogenesis, repair nerve damage, and provide new treatments for cerebral injury and neurodegenerative diseases, such as Alzheimer's disease (AD), Parkinson's disease (PD), and strokes [176]. Here, we review the effects and underlying mechanisms of Chinese medicinal compouds, single herbs, and herb extract/the Chinese herbal monomer on NSCs proliferation and differentiation. Summarized are shownin Tables 3 and 4.

### 3.1 Effects of compound Chinese medicinal preparation on NSCs proliferation and differentiation

Experimental studies have found that compound Chinese medicine preparation has an important regulatory role on NSCs proliferation and differentiation. The compound prescriptions mainly include Huoxue Huayu (promoting blood circulation and removing blood stasis) and tonifying kidney recipes, of which Buyang Huanwu Decoction is a classic TCM prescription. This can promote blood circulation and dredge the meridians, and thus often used for the treatment of cerebrovascular disease. Buyang Huanwu is composed of Astragalus membranaceus (Fisch.) Bge (120 g), Angelica sinensis (Oilv.), Diells (10 g), Paeonia lactiflora Pall (10 g), Ligusticumwallichii Franch (10 g), Carthamus tinctorius L (10 g), Semen Persicae (10 g) and Flos carthami (4.5 g). Recent research has demonstrated that Buyang Huanwu Decoction can promote the proliferation and differentiation of NSCs, and improve the expression of growth-associated protein-43 (GAP-43) [177]. It can also promote the differentiation of neuroepithelial stem cells into neurons and astrocytes [178], the growth of nerve cells and nerve fibers, and the growth and differentiation of NSPCs. Experimental results have demonstrated that the content of Ca2^+^significantly decreased and expression of neurofilament (NF) and glial fibrillary acidic protein (GFAP) significantly increased in the NSPCs treated with Buyang Huanwu Decoction [179].

Jiaweisinisan consists of Stellaria dichotoma L. var. lanceolata Bge, Paeonia lactiflora Pall, Citrus reticulata Banco, Poncirus trifoliate (L.) Raf, Lycium barbarum L, Gardenia jasminoides Ellis, Radix Rehmanniae, and Abalone. These are weighed according to the ratio of 1:3:1:3:1:4:6, respectively. This Jiaweisinisan prescription can also promote the proliferation of hippocampal NPCs, and inhibit the apoptosis of glial cells and neurons differentiated from Hippocampal-NPCs [180].

Shengyu decoction, a traditional Chinese medicine, has been used to treat diseases that involve a deficit in “qi” and “blood.” Modified Shengyu decoction (MSD) was designed to treat brain injury after head trauma, according to traditional Chinese medicine theories, and is based on the traditional Shengyu. Four additional herbs are in the MSD: Salvia mil-tiorrhiza Bunge, Commiphora myrrha (Nees) Engl., Acorus calamus L, and Curcuma aromatica Salisb. A study on the treatment of traumatic injury in rats using the Shengyu decoction showed that it could increase the expression of nerve growth factor (NGF), glial cell line-derived neurotrophic factor (GDNF), neural cell adhesion molecule (NCAM), and Tenascin-C (TN-C) in the cortex and hippocampus of rats. It can also inhibit the expression of Nogo-A and promote the proliferation of NSCs/NSPCs and their differentiation into neurons [181].

Fuzhisan is a traditional Chinese medicine prescription, composed mainly of four Chinese medicinal herbs: Panax ginseng C. A. Mey, Scutellaria baicalensis Georgi, Acorus gramineus Soland, and Glycyrrhiza uralensis Fisch. Experimental studies have shown that Fuzhisan can promote the proliferation of NSPCs and improve the survival rate of newborn cells [182].

Xiehuo Bushen Decoction consists of Rheum officinale baill, Paeonia suffruticosa Andr, Paeonia lactiflora Pall, Astragalus membranaceus (Fisch.) Bge, Cuscuta Lam, Viscum coloratum (Kom.), and Nakai. It can promote the survival and differentiation of NSCs transplanted into a brain after cerebral hemorrhage. The possible mechanism is that the Xiehuo Bushen Decoction can enhance the expression of interleukin 4 (IL-4) mRNA and down-regulate the expression of interferon-gamma (IFN-gama) mRNA [27].

Polygonummultiflorum Thunberg complex composition-12 (PMC-12), a mixture of four medicinal herbs, includes Polygonum multiflorum Thunb, Radix Polygalae, Rehmannia glutinosa, and Acorus gramineus Soland. PMC-12 was found to promote the proliferation of NSPCs in the hippocampus, increase the survival rate of newborn neurons, and encourage neurogenesis in the hippocampus [183].

Although compounds of Chinese medicine have been found to regulate the proliferation and differentiation of NSCs through experiments, it has not been determined whether one or a combination of the active ingredients influences the proliferation and differentiation of the NSCs, because of the complexity of the active ingredients of the compounds of Chinese medicine. Further experiments are therefore required to establish this.

### 3.2 Effect of single herbs on NSCs proliferation and differentiation

Extensive research has been conducted on Chinese herbs, such as those used for Huoxuehuayu and Qingrejiedu (clearing away heat and toxic material) in the study of NSC proliferation and differentiation.

Salvia miltiorrhiza Bge is a common traditional Chinese herb for Huoxuehuayu. It has anti-oxidation and anti-inflammatory functions and is often used to treat nervous system diseases. Studies have shown that Danshen can increase the expression of the nestin significantly, promote the differentiation of induced multifunctional NSCs into neurons *in vitro*, and promote the survival and differentiation of NSCs derived from multifunctional stem cells [22]. Another commonly used herb for Huoxuehuayu, Sambucus williamsii Hance, was also shown in *in vitro* experiments to promote the proliferation of NSCs, but the study was conducted together with the herbs of Qingrejiedu [184].

The herbal preparation composed of Scutellariacalensis Georgi, Phellodendron chinense Schneid, and Ligusticumwallichii Franch has been shown to promote the proliferation of NSCs *in vitro. In vitro* study shows that this herbal medicine can improve the symptoms of depression in mice models, which was mainly achieved through the machanism of increasing the content of corticosterone and promoting hippocampal precursor cell proliferation [23].

Experiments also showed that the traditional Chinese herb Sambucus williamsii Hance can promote the differentiation of induced pluripotent stem cells (iPSCs) into neurons by up-regulating the expression of tubulin-1 (Tuj1) and nestin, and down-regulating the expression of Oct4 and Sox2 [184].

### 3.3 Effects of Chinese herbal monomer on NSCs proliferation and differentiation

**Figure 1.**
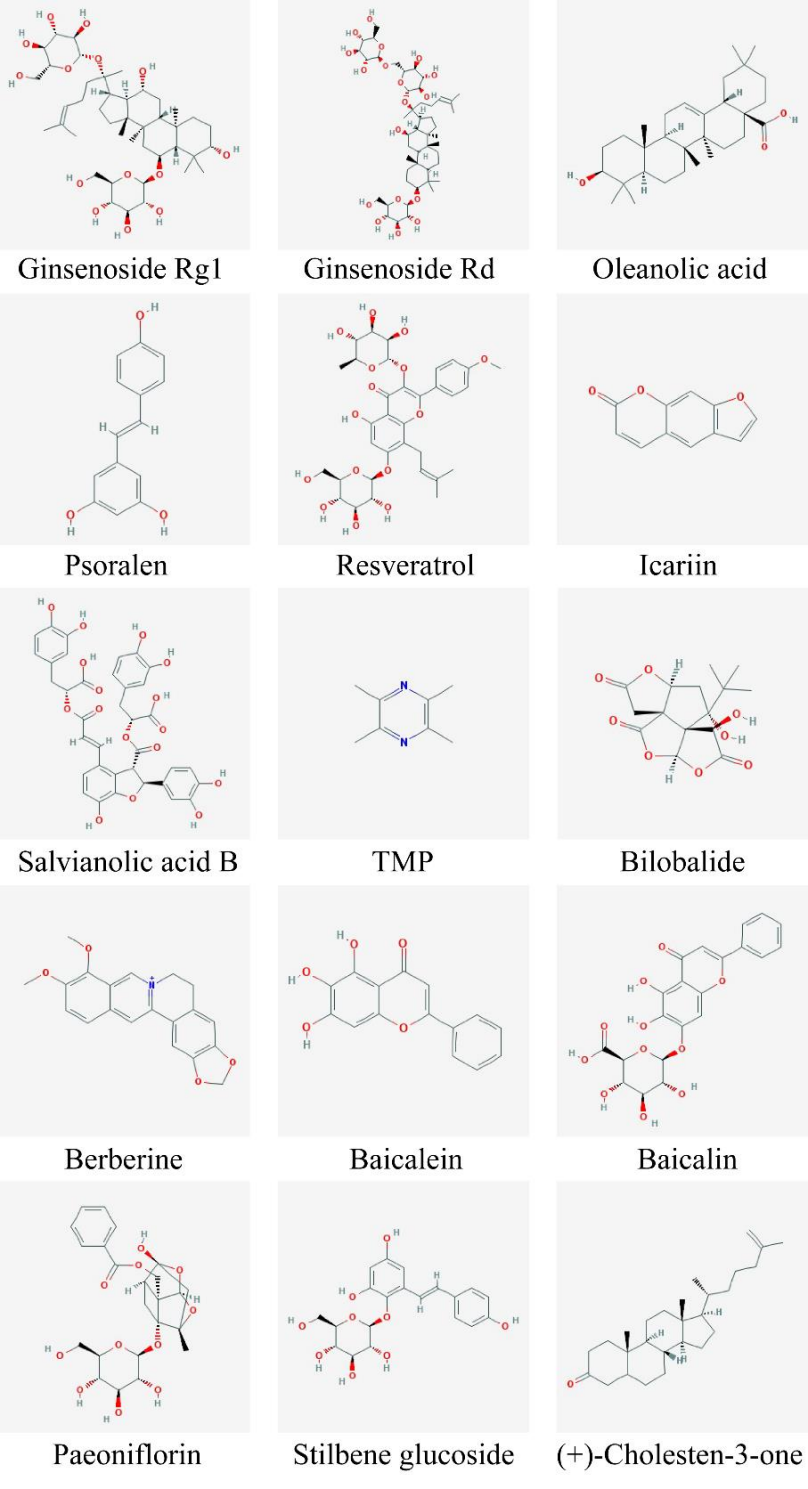
Structures of Chinese herbal monomers

In recent years, numerous experiments have demonstrated that the extract of Chinese herbal monomer Plays a specific regulatory role in NSC proliferation and differentiation. The current research mainly focuses on the extraction of effective components from the Chinese tonifying herbs HuoxueHuayu and QingreJiedu (Fig. 1).

#### 3.3.1 Monomers from Chinese herbs

##### 3.3.1.1 Tonifying Qi herbs

Panax ginseng C. A. Mey is an herb used in TCM for tonifying “Qi”. Recent research has shown that it has roles in anti-anxiety, anti-depression, and cognitive function-enhancing, among others. The numerous active ingredients in Panax ginseng C. A. Mey have the effect of nerve protection [185]. Ginsenoside Rg1 can promote the differentiation of NSCs into neurons and play a neuroprotective role by increasing the expression of SOX-2 and decreasing the expression of interleukin 1β (IL-1β), interleukin 6 (IL-6), and TNF-a. The differentiation of NSCs can be promoted by cAMP protein kinase A (PKA) and PI3K-Akt signaling pathway [186, 187]. Ginsenoside Rd can promote the proliferation of NSCs by regulating the expression of neurotrophic factor 3 and activating the expression of inducible nitric oxide synthase (iNOS) and N-methyl-D-aspartic acid (NMDA) receptors [188].

##### 3.3.1.2 Tonifying Yin herbs

Oleanolic acid extracted from the “Yin”-tonifying Chinese herbal Ligustrum lucidum Ait and stilbene glycoside extracted from Fallopia multiflora (Thunb.) Harald have been found to promote the self-renewal and differentiation of NSCs *in vitro*. The relevant mechanism may be the increase of the expression of tubulin and the ratio of tubulin/DAPI [24].

Resveratrol is a polyphenolic compound found in various plants, and is an effective component of the Chinese herb Fructus Mori. Experiments have established that it can promote the survival and proliferation of NSCs. In addition, the expression of Patched-1 (Ptch-1), Smoothened (Smo), Gli-1 protein, and RNA were all up-regulated in resveratrol-treated NSCs [25, 158].

The tortoiseshell cholesterol extracted from Chinemys reevesii (Gray), (+)-Cholesten-3-one, can induce NSCs into dopaminergic neurons through a bone morphogenetic protein (BMP) signal, thus providing a possible new treatment for PD [189].

##### 3.3.1.3 Tonifying kidney herbs

Psoralen is an effective component extracted from the Chinese herb (Psoralea corylifolia L.), and is representative of kidney-tonifying herbs. Psoralen has been found to increase the expression of glial fibrillary acidic protein (GFAP) in NSCs *in vitro*, thereby promoting the differentiation of NSCs into astrocytes [190]. Icariin, the extract of Epimediumgrandiflorum Morr, which is another well-known Chinese herb for kidney tonifying, can promote self-renewal and differentiation in NSCs. This can be mediated by the related kinase signal transduction pathways [191].

#### 3.3.2 Huoxuehuayu herbs

Salvianolic acid B is the root and rhizome of Salvia miltiorrhiza Bge, a traditional Chinese herb of HuoXueHuaYu. It can maintain the self-renewal of NSCs/ progenitor cells and promote the proliferation of NSCs though the regulation of the PI3K/Akt signaling pathway [192]. It also promotes the growth of the synapses of NSCs and the differentiation of neurons [193].

Tamethylpyrazine extracted from Ligusticum wallichii Franch can promote the proliferation and differentiation of NSCs under the condition of hypoxia *in vitro* [194], and also promotes the differentiation of NSCs after cerebral ischemia [195].

Panax notoginseng saponins (PNS), which has the effect of Huoxuehuayu, simultaneously promotes the expression of nestin/BrdU, improves the expression of tubulin-1 (tuj1), vimentin, and Nestin mRNA, and promote the self-renewal, proliferation, and differentiation of NSCs [196].

Bilobalide extracted from Ginkgo biloba can increase the phosphorylation of the Cyclic AMP response element binding protein (CREB) in NSCs and the level of the neurotrophic factor, and promote the proliferation of NSCs [197].

#### 3.3.3 Qingrejiedu herbs

Berberine is the main active ingredient of Coptis chinensis Franch, a traditional Chinese herb of Qingrejiedu. Studies show that it can inhibit cell cycle arrest and promote the survival and differentiation of NSCs [198]. Baicalein and baicalin are the two main active components of Scutellaria baicalensis Georgi, and baicalein can promote the differentiation of NPCs into neurons. The mechanism may be related to the increase of the expression of presynaptic protein, synapsin I and postsynaptic density proteins 95 (PSD95) [199]. Baicalein can also inhibit apoptosis and promote the differentiation of NSCs [200]. Baicalin, another extract, can determine the fate of NSCs, and promote neurogenesis [201]. It also can regulate the expression of phosphorylated signal transducer transcription3 (p-stat3) and bHLH family proteins, subsequently promoting the differentiation of NSCs/NSPCs [202].

Paeoniflorin is a natural compound extracted from the roots of the Chinese herbs Paeonia lactiflora Pall and Paeonia suffruticosa. It can promote the proliferation and survival of NSCs and precursor cells *in vitro* and inhibit the apoptosis of cells. The mechanism may be related to down-regulation of the expression of inhibitor Κb (iκB), nuclear transcription factor-κB (NF-κB), and interleukin-1β (IL-1β)[203].

As mentioned above, the natural compounds extracted from traditional Chinese medicine, which play a regulatory role in the proliferation and differentiation of NSCs, can be divided into the three categories of tonifying (for tonifying Qi, Yin, and kidney), Huoxuehuayu, and Qingrejiedu herbs. The effects of tonifying herbs on NSCs proliferation and differentiation have been studied most. In the theory of TCM, the nerve function damage is often due to kidney deficiency caused by the deficiency of the marrow-reservoir. These tonifying herbs are therefore often used for the treatment of nervous system diseases. In addition, kidney essence is also believed to be critical in maintaining a variety of life activities. The potential function of various organs can be stimulated through the method of tonifying the kidneys, which is interlinked with the method of promoting the proliferation and differentiation of NSCs to repair brain neurons in the treatment of brain damage and neurodegenerative diseases. In addition, the insufficient cerebral blood supply caused by cerebral arteriosclerosis or atherosclerotic plaque inflammatory response is also a common cause of nervous system diseases. Therefore, the heat detoxification practice of TCM is also often applied to treat nervous system diseases, including the clinical use of Huoxuehuayu and Qingrejiedu herbs. Experimental studies also demonstrate that Chinese herbs and their active ingredients can regulate the proliferation and differentiation of NSCs, which provides a broader space for discovering drugs that can regulate the proliferation and differentiation of NSCs.

## 4. Summary and Perspectives

NSCs pass through the different stages of NSPCs, NPCs, and neuroblasts in the process of proliferation and differentiation into neural cell lineage, and their corresponding markers are found at different stages. In the process of neurogenesis, and in NSC proliferation and differentiation, a variety of internal and external factors precisely regulate NSCs through different protein pathways. Studies have found that single herbs, herb extracts/Chinese herbal monomers, and compounds of Chinese medicine have a certain regulatory role on the proliferation and differentiation of NSCs. However, most of the current studies focus on single pathways. However, the regulation of the NSC proliferation and differentiation is involved in a complex signal network. In addition, NSC research into TCM lacks multi-targeted and multi-channel approaches, which is a systematic deficiency and thus they cannot fully explain the detailed mechanisms underlying regulating NSC by TCM. The effects of TCM promoting neurogenesis are still in the experimental stage, and may not be ready for the clinical application. In short, TCM should be played to its advantages, and in combination with modern medicine used to explore its potential in the regulation of neurogenesis to provide new possibilities for the treatment of brain damage and neurodegenerative diseases.
